# A problem of overlap

**DOI:** 10.1017/S0952523814000340

**Published:** 2015-04-23

**Authors:** ROGER B. TOOTELL, CESAR ECHAVARRIA, SHAHIN NASR

**Affiliations:** 1 Athinoula A. Martinos Center for Biomedical Imaging, Massachusetts General Hospital, Charlestown, Massachusetts; 2 Department of Radiology, Harvard Medical School, Boston, Massachusetts

**Keywords:** fMRI, LOC, MT, MTc, Visual cortex

## Abstract

Here we propose that earlier-demonstrated details in the primate visual cortical map may account for an otherwise puzzling (and problematic) finding in the current human fMRI literature. Specifically, the well-known regions LO and MT(+) reportedly overlap in the human cortical visual map, when those two regions are localized using standard stimulus comparisons in conventional fMRI experiments. Here we describe evidence supporting the idea that the apparent functional overlap between LO and MT arises from a third area (the MT crescent: “MTc”), which is well known to surround posterior MT based on earlier histological, neuroanatomical, and electrophysiological studies in nonhuman primates. If we assume that MTc also exists in human visual cortex, and that it has a location and functional properties intermediate to those in LO and MT, simplistic modeling confirmed that this arrangement could produce apparent overlap between localizers for LO and MT in conventional fMRI maps in human visual cortex.

## Introduction

“How can you be in two places at once when you're not anywhere at all?”

The Firesign Theater, 1969.

Previous studies have reported that two of the most well-established visual cortical areas overlap in the cortical map, when defined with independent functional localizers (Denys et al., 2004; Kolster et al., 2010; Kolster et al., 2014). Those reportedly overlapping areas are (1) the motion-selective area MT+ and (2) the object-selective lateral occipital (LO) region. Such functional overlap conflicts with the standard expectation that the data arise from two distinct and fixed cortical visual areas.

Several possible explanations arise for this overlap. One possible explanation would arise from simple differences in the threshold of activity, and/or inter-subject variation. However, such explanations may not fully account for all of the data. Here we propose that the region of apparent overlap may instead (or in addition) reflect the presence of a third, independent cortical visual area (“MTc”, initially known as “V4t”). The existence and topography of this third area have been well known for many years in studies of nonhuman primates (e.g., Tootell et al., 1985; Desimone & Ungerleider, 1986; Gattass et al., 1988; Krubitzer & Kaas, 1990; Kaas & Morel, 1993; Malach et al., 1997), and histological evidence suggests its presence in humans (Tootell & Taylor, 1995). However, MTc has not been previously incorporated into the current literature from human neuroimaging, to our knowledge.

### Background, area MT (V5)

A main component in human area MT+ (Zeki et al., 1991; Watson et al., 1993; Tootell et al., 1995*b*) is the presumptive human homologue of motion-selective area MT (also known as V5), which has been demonstrated in essentially all primates tested (e.g., Allman & Kaas, 1971; Dubner & Zeki, 1971; Allman et al., 1973; Maunsell & Van Essen, 1983*b*; Albright et al., 1984; Felleman & Kaas, 1984; Krubitzer & Kaas, 1990; Tootell & Taylor, 1995). In humans, the addition “+” in “MT+” tacitly acknowledges the presence of additional (but incompletely defined) functionally similar areas neighboring area MT. One of these neighboring areas is the presumptive human homologue of MST (Huk et al., 2002); other subdivisions of MT+ have been based on retinotopic criteria (Kolster et al., 2010; Kolster et al., 2014). In nonhuman primates, MST typically borders MT on the anterior side, so it is not relevant for the results here; instead we focus on the posterior border region of MT.

In all nonhuman primates tested, area MT *per se* is oval-shaped and small relative to V1/V2 in the cortical map. MT is also located remote from, and roughly anterior to, the confluent foveal representation of retinotopic areas V1/V2. Based on histological (Tootell & Taylor, 1995) and neuroimaging evidence (e.g., Zeki et al., 1991; Watson et al., 1993; Tootell et al., 1995*a*; Tootell et al., 1995*b*), human MT has similar attributes. Histologically, MT in both human and nonhuman primates shows distinctive staining for myelin (Van Essen et al., 1981), cytochrome oxidase (Kaas & Morel, 1993; Tootell & Taylor, 1995; Malach et al., 1997), and the monoclonal antibody CAT-301 (Hockfield et al., 1990; Tootell & Taylor, 1995). CAT-301 is linked to magnocellular stream subdivisions at lower levels including V1 (Hockfield et al., 1990), from which MT receives strong input (e.g., Maunsell & Van Essen, 1983*a*; Maunsell et al., 1990).

Based on neuroimaging results, the core of human area MT+ is also similar to that found in monkeys, insofar as that can be determined across the two species. In early electrophysiological recordings from monkeys, many neurons in MT (and neighboring areas) respond selectively to visual stimuli that are moving in a given direction; i.e., those neurons respond selectively to moving stimuli (*vs*. stationary) and more specifically, to a specific range of stimulus direction (*vs*. other directions) (Dubner & Zeki, 1971; Maunsell & Van Essen, 1983*b*; Maunsell et al., 1990). In fMRI acquired from awake fixating monkeys and humans, such motion- and direction-selective properties are also a defining functional property of MT and MT+.

Partly because of this wide range of distinguishing features, MT(+) was one of the first extrastriate areas distinguished in the cortical visual map in both nonhuman (Allman & Kaas, 1971; Dubner & Zeki, 1971) and human (Zeki et al., 1991; Watson et al., 1993; Tootell et al., 1995*b*) primates. MT(+) has been demonstrated in essentially all primates tested, including prosimians (Allman et al., 1973; Krubitzer & Kaas, 1990), New (Fiorani et al., 1989; Krubitzer & Kaas, 1990), Old World (Van Essen et al., 1981; Tootell et al., 1985) monkeys, and humans (Tootell & Taylor, 1995). Thus area MT(+) is presumably a fundamental component of the visual cortical circuitry in many or all primates. Scattered data suggest that a MT homologue may also exist in some nonprimate mammals, including cats (Payne, 1993) and squirrels (Paolini & Sereno, 1998).

In human neuroimaging studies, MT+ has typically been localized using a stimulus contrast of moving *versus* stationary stimuli: the moving stimuli elicit a relatively higher response in MT+. More recent imaging studies have also localized area MT+ based on its high level of myelination (Glasser & Van Essen, 2011; Lutti et al., 2014) and cytoarchitecture (Malikovic et al., 2007).

### Background, LO region

The second of these overlapping regions, the LO complex, is well established in humans. Human LO is thought to respond selectively to isolated images of objects, *versus* various control versions of non-objects. The most common type of control stimuli are grid-scrambled images of the same objects. This has become a standard “localizer” for human LO (Malach et al., 1995; Grill-Spector et al., 1998). Although literally hundreds of fMRI studies have measured activity in human LO, there has been little systematic study of the presumptive macaque homologue of human LO based on similar stimuli (but see Sawamura et al., 2005; Tsao et al., 2003; Denys et al., 2004).

This interpretation of LO activity is mid-level in nature, as opposed to the more feature-selective responses found in lower tier areas, and the more category-selective responses reported at presumptively higher tier areas. Notably, this typical intact-*versus*-scrambled objects localizer activates a wide swath of visual cortex extending over a large region of lateral visual cortex. This swath of activity is located anterior to the lower-tier, retinotopically-definable areas (e.g., V1, V2, V3, and V4). However, since the initial report, it has been clarified that many of the cortical regions found within that wide swath of “object selective” activity respond more selectively to more discrete categories of visual stimuli. Such areas include the “place selective” parahippocampal place area (PPA) (Epstein & Kanwisher, 1998; Nasr et al., 2011) and transverse occipital sulcus (TOS) (Grill-Spector, 2003), the face-selective fusiform face area (FFA) (Kanwisher et al., 1997; Rajimehr et al., 2009), and perhaps the posterior fusiform sulcus (pFS) (Grill-Spector et al., 1999). Accordingly, these distinct cortical areas are now interpreted as areas distinct from “LO”. After excluding these areas, the remaining “core” of LO is typically drawn immediately posterior to the posterior ∼half of MT, and anterior to human V4 (Malach et al., 1995; Halgren et al., 1999; Tootell et al., 2003).

### Evidence for overlap

**Fig. 1.**
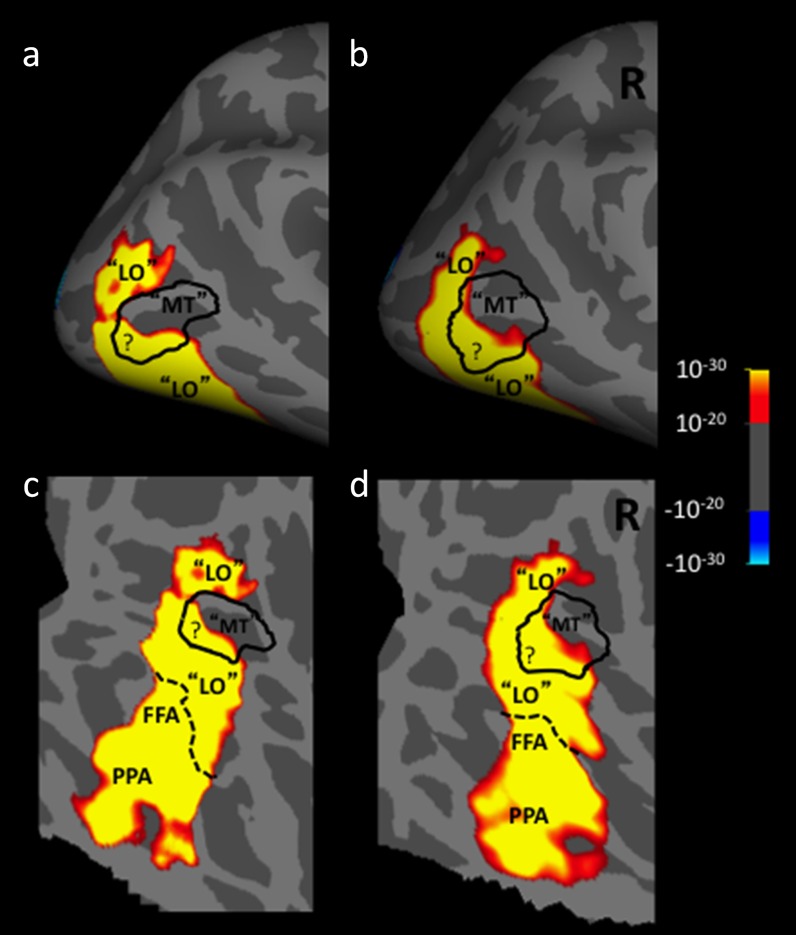
Empirical group average data for 13 subjects. The activity map shows higher fMRI activity in response to presentation of intact single isolated objects, compared to grid-scrambled versions of those same stimuli. This stimulus contrast is a conventional localizer for LO. The black solid outline indicates the border of MT+, localized conventionally by presentation of moving *versus* stationary rings. The areas are enclosed in quotes because the exact borders of these areas are called into question by the functional overlap indicated in the map by “?”. The two stimulus contrasts were acquired from independent runs. Panels. (**a** and **b**) Lateral view of a group average (*n* = 13), showing activity in the posterior cortex, in a surface-inflated format. In all panels, light gray denotes gyri and dark gray denotes sulci. (**c** and **d**) Flattened view of the same averaged brain and activity. The dotted line shows the border of the pFS. Below the dotted black line are cortical areas that are selective for different stimulus properties, i.e., faces (FFA) and scenes (PPA). For ease of comparison, the left hemisphere (**a** and **c**) has been reversed left-*versus*-right.

When the borders of these two areas are based on their empirical functional localizers (rather than drawings thereof), two overlapping regions result (Fig. 1). In other words, the activity generated by the intact-*versus*-scrambled object localizer in the core region of LO can overlap in topography with the activity generated independently by the moving-*versus*-stationary localizer of MT+. This overlap between LO and MT has been formally reported (Denys et al., 2004; Kolster et al., 2010; Kolster et al., 2014), and Fig. 1 illustrates such overlap from a fMRI group map (*n* = 13) generated in our own laboratory. In the latter dataset, the LO/MT+ overlap includes posterior and ventral regions of MT+. Of course, the extent of such spatial overlap depends on the scanner sensitivity, the number of acquisitions per subject, and on the statistical threshold chosen. However, in the group map of Fig. 1, the overlap reached 12 mm across the cortical map.

One conceivable interpretation for this unexpected overlap is that different subdivisions of LO have different functional properties. A similar interpretation can be made for area MT+. However, such a functional subdivision would be highly unusual; typically a given functional property (e.g., orientation sensitivity in V1) applies uniformly throughout a given cortical visual area, within certain limits. In contrast, proposals that attribute differing functions to two topographically related areas (i.e., VP and V3) are counterintuitive and uncommon, as acknowledged elsewhere (Burkhalter et al., 1986).

Because the spatial resolution of conventional neuroimaging techniques is known to be limited, another possibility is that this LO/MT+ overlap is apparent rather than biologically real. By this interpretation, *apparent* overlap could arise using neuroimaging techniques that have inadequate spatial resolution to clearly distinguish the border. For instance, BOLD-based fMRI at conventional spatial resolution (i.e., 3 mm^3^), and at low activity thresholds, could produce some spatial overlap between even well-understood cortical areas that border each other (e.g., V1 and V2). However, empirically, the extent of spatial overlap found between human MT and LO often exceeds that seen between other cortical areas (e.g., V1 and V2). This overlap also exceeds the extent that could be logically expected from the factors listed above. Finally, this interpretation cannot explain the presence of significant overlap on one side of MT, without overlap on the other side (Fig. 1).

### A third, intervening area

Here we propose that the spatial overlap between these two important areas (MT+ and LO) might be explained by recalling a third, intervening area which is located between LO and MT+. Here we refer to that area as “MTc”, although it has had different names as its properties were progressively clarified (see below).

This area was initially named “V4t” (i.e., “V4 transition”), because it was thought to be an area with properties intermediate between those found in V4 and the adjacent MT, in macaque monkeys. Histologically, V4t was reported to show a level of myelination that was intermediate relative to the high myelination in the lower layers of area MT, and the light myelination found in area V4 (Desimone & Ungerleider, 1986; Kaas & Morel, 1993). Electrophysiologically, area V4t showed an intermediate number of direction selective cells, compared to the higher percentage of such cells in MT, and the lower percentage in V4 (Desimone & Ungerleider, 1986). However that initial description of V4t was tentative, because both the electrophysiology and the myelin staining could have arisen by noise in the localization of the border between macaque MT *versus* V4. Related caveats were expressed for distinguishing between MTc and MTl (Rosa & Elston, 1998).

Further reports of a retinotopic re-representation of the visual field in V4t (independent of the representation found in adjacent area MT) suggested that V4t was an independent visual area, not a “transition” area between MT and V4 (Desimone & Ungerleider, 1986; Gattass et al., 1988). Additionally, V4t was found to have independent neuroanatomical connections, compared to the pattern of connections in either V4 or MT (Desimone & Ungerleider, 1986; Krubitzer & Kaas, 1990; Kaas & Morel, 1993; Malach et al., 1997).

**Fig. 2.**
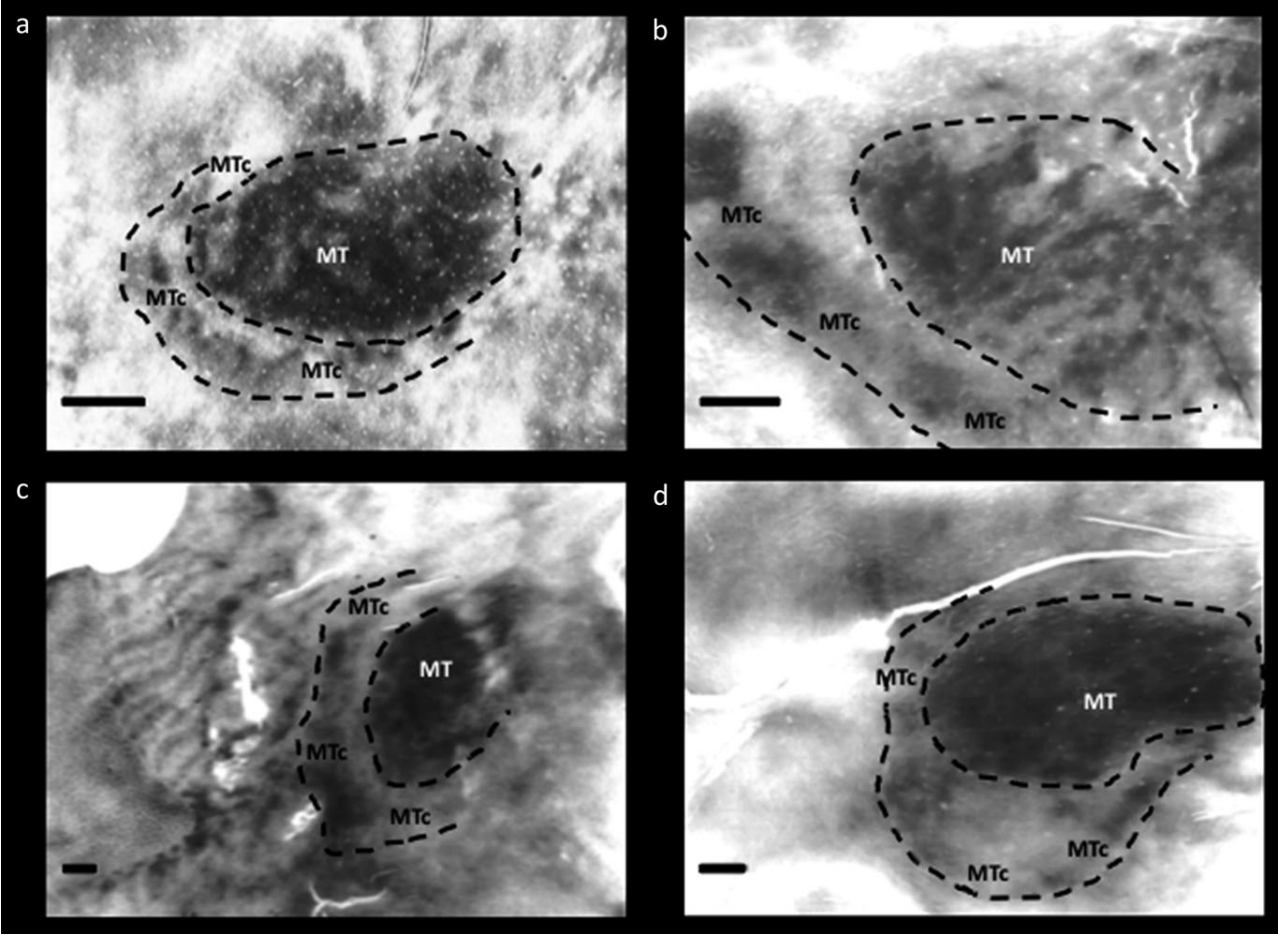
Cytochrome oxidase (CO) staining of area MT and MTc in various primate species: (**a**) owl monkey (*Aotus trivirgatus*), (**b**) green monkey (*Chlorocebus sabaeus*), (**c**) macaque monkey (*Macaca mulatta*), and (**d**) humans. Images modified from Tootell et al. (1985) and Tootell and Taylor (1995). All scale bars = 2 mm.

Further evidence from histology strengthened the conclusion that “V4t” is in fact a distinct cortical area, interposed between V4 and MT. This evidence was initially based on staining patterns for cytochrome oxidase (CO) in physically flattened cortex (Tootell et al., 1985; Krubitzer & Kaas, 1990; Kaas & Morel, 1993; Tootell & Taylor, 1995) (see Fig. 2). Specifically, this region has an outer band that stains darkly for CO, and a band of lighter CO staining between MT and the darker outer band. The outer boundary of this area is ovoid in topography, effectively wrapping around the posterior ∼half area MT; thus it is generally located between V4 and MT – like the topography previously attributed to “V4t”. This CO “band” has been documented in five widely separated primate species: two New World species (Allman & Kaas, 1974; Rosa & Elston, 1998), two Old World nonhuman primates (Desimone & Ungerleider, 1986; Tootell & Taylor, 1995), and humans (Tootell & Taylor, 1995) (see Fig. 2). Based on this histological evidence, this area was initially called the “MT ring” (Tootell et al., 1985). Subsequently the name was corrected to the “MT crescent” (MTc) because the region is less evident around the peripheral vertical meridian representation in MT, compared to the foveal representation of MT (Allman & Kaas, 1974). In the owl monkey, the cortical region located immediately posterior to MT (“DLa”) includes a thin mirror-image representation of the visual field (Sereno et al., 1994). Based on its location and width in the cortical map, this retinotopic representation likely coincides with area MTc. Based on histology, MTc appears to be relatively wider in humans (2–6 mm wide), compared to the MTc in the cortical map of nonhuman primates (1–4 mm wide) (Tootell & Taylor, 1995) (see Fig. 2 [included above]).

Many (perhaps most) cortical visual areas have distinctly different connections compared with those in neighboring cortical areas; this is one main way that cortical areas are defined. In addition, distinct subregions within a given area sometimes have their own connections to subregions in other areas (e.g., from V1 blobs to V2 stripes; Livingstone & Hubel, 1988; Sincich & Horton, 2002). In both cases, such differences in connections reflect differences in the anatomical and functional streams in visual cortex. Consistent with this general finding, the darkly labeled patches in outer MTc (but not the inner, lightly stained stripe) are connected with MT (Krubitzer & Kaas, 1990; Kaas & Morel, 1993; Malach et al., 1997).

We assume that the electrophysiologically defined “V4t” corresponds to the histologically defined “MTc”, for several reasons. Mainly, V4t and MTc are located in the same cortical location, insofar as they both have been mapped; i.e., both are bands that encircle posterior/ventral MT. Here we speculate further that the direction selective neurons reported earlier in “V4t” are located in the outer, CO-dark bands within MTc. This speculation is based on the fact that MT itself shows distinctively higher CO activity, compared to neighboring cortical areas, including V4. This assumption of higher motion/direction selective activity in MTc is also supported by the fact that the connections from the direction-selective MT to MTc are concentrated on the CO-dark (rather than the CO-light) regions of MTc (Krubitzer & Kaas, 1990; Kaas & Morel, 1993; Malach et al., 1997). In any event, the latter assumption (that direction selective cells are preferentially localized in the outer dark CO band of MTc) is not crucial for our model of the empirical fMRI results (below).

Based on this evidence, it could be argued that the dark and light CO-labeled bands in MTc could be named differently (e.g., MTc alpha and MTc beta). However for simplicity, here we refer to the pair of bands using the single name “MTc”.

Presciently, Desimone and Ungerleider had speculated about a similar organization: “V4t [here, reinterpreted as MTc] could be considered to be a strip within V4, with its own unique myeloarchitecture, neuronal properties, and anatomical connections”.

### Proposal/hypothesis

**Fig. 3.**
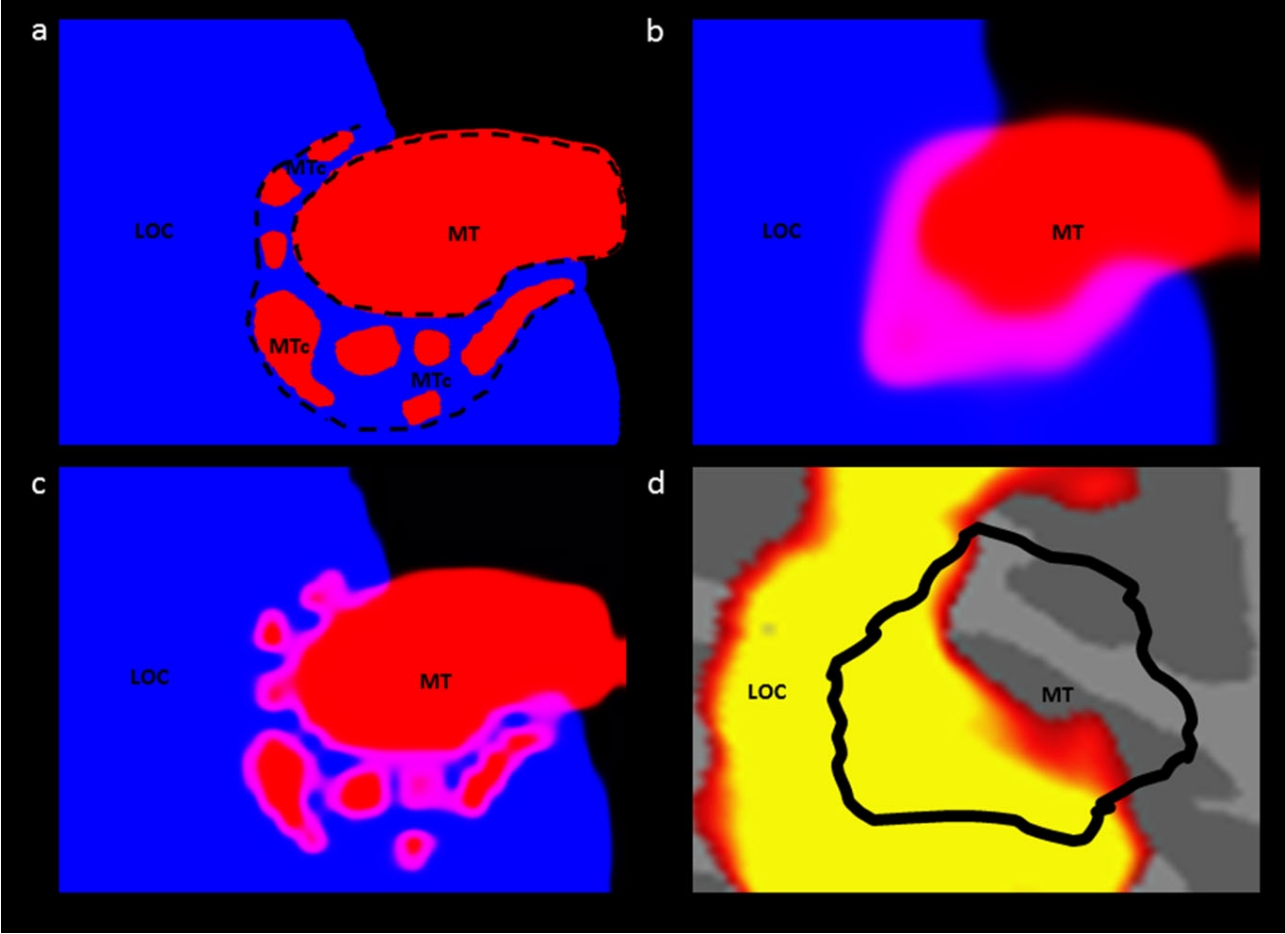
Proposed source of apparent overlap. (**a**) Schematic “real” topography of areas LOC, MTc, and MT, based on CO staining data from humans. The map was generalized from Fig. 2d, in a right hemisphere. (**b**) Panel **a**, after spatially filtering to approximate conventional fMRI scanning resolution (3 mm^3^). The image was first down-sampled to a resolution of 1 pixel = 3 mm^2^ then the luminance of the red and blue channels was scaled with a sigmoid function. The resulting image shows the resulting apparent overlap of the 2 areas in purple. Additional “blurring” may arise due to hemodynamic factors and analytic filtering. (**c**) Simulation of the effect of high fMRI resolution (1 mm^3^) on the schematic map in panel **a**. The prediction is that at this higher resolution one would be able to observe the motion-selective patched in MTc. (**d**) Empirical data from Fig. 1d enlarged to the same scale of the model, for comparison. The modeling data (panel **b**) match the empirical data (panel **d**) fairly well.

Given the evidence discussed above, we propose that the apparent spatial overlap in fMRI signals that is currently attributed to “LO” and “MT+” arises from MTc, as follows. First, we propose that the motion-biased response in the “MT localizer” (i.e., moving *vs*. stationary stimuli) arises from both MT in addition to the outer (dark CO) band of MTc. Secondly, the “object selective” responses (objects *vs*. scrambled objects) arise from the classic LO core in addition to the nearby low-CO band in MTc. Third, all patterns are “blurred” by the known spatial limitations of the fMRI, including minor misalignment in group maps. The result is an apparent spatial overlap between MT and LO as defined by their respective localizers. This proposal is illustrated in Fig. 3.

### Experimental validation

In future studies, there are several possible experimental approaches to test this hypothesis, all requiring relatively high spatial resolution to distinguish human MTc (and its paired subbands) from LO and MT+. Such possible approaches would include a spatial resolution of less than 1 mm^3^), with high sensitivity (e.g., with a 7T scanner), plus extensive signal averaging. Though such an experiment would be technically challenging, this resolution is within the spatial limits of fMRI, at least with higher-field scanners (e.g., Cheng et al., 2001; Moon et al., 2007; Yacoub et al., 2008; Swisher et al., 2010; Polimeni et al., 2010; Zimmermann et al., 2011).

Given this higher spatial resolution, one functional way to distinguish these areas would be compare the activity maps in response to the two localizing stimuli (i.e., intact *vs*. scrambled objects, and moving-*vs*.-stationary stimuli) (e.g., Fig. 3). The expectation would be that the motion-selective patches of MTc would be revealed simply by increases in spatial resolution. Related ways to distinguish these areas would be to test for the re-representation of the visual field reported in V4t (Desimone & Ungerleider, 1986; Gattass et al., 1988; see also Kolster et al., 2014). In addition, it might be helpful to be able to resolve the high myelination that underlies MT, which is not present in MTc (Tootell et al., 1985; Kaas & Morel, 1993).

### Additional considerations

For simplicity, the above account describes a single possibility, involving MT, MTc, and LO, and related functional properties (e.g., direction selectivity, intrinsic activity differences). However, additional fMRI results from humans may have also activated one or more of the regions we discuss, in a different experimental context. Because functional properties specific to MTc remain largely unknown, any such additional interpretations could complement (rather than conflict with) the MTc interpretation described above.

For instance, population receptive field (pRF) mapping has described two full hemifield representations within MT+ (Amano et al., 2009). These regions, labeled TO-1 and TO-2, lie just anterior to the already known retinotopic maps within LO (i.e., LO-1 and LO-2). Post-hoc comparison of the retinotopically defined regions with conventional localizers suggests that the intact-*versus*-scrambled localizer evokes activity within LO-2, the motion-*versus*-static localizer evokes activity within both TO regions, and an MST localizer (motion *vs*. static only in ipsilateral hemifield) evokes activity only within TO-2. A more detailed characterization of MTc is needed to determine the relationship between this area and the TO retinotopic maps.

It could also be argued that the functional overlap (e.g., Fig. 1) arises from functionally specific patches, i.e., portions of retinotopically defined areas, rather than from whole areas *per se*. In macaques, patches within V4 have been reported to be selective for stimulus color (Tootell et al., 2004; Conway et al., 2007; Tanigawa et al., 2010), orientation (Tanigawa et al., 2010), and/or curvature (Yue et al., 2014). Thus in humans, it is conceivable that uncontrolled variables in the object- or motion-defined localizers produced biased responses in such patches, leading to apparent functional overlap in LO (adjacent to retinotopically defined human V4). However within macaque V4, the exact locations of such V4 patches may vary across individuals (Tootell et al., 2004; Conway et al., 2007; but see Yue et al., 2014). If so, such patches would not be aligned in the group map, and thus could not easily account for the current overlap.

Finally, it has been reported that images of body parts selectively activate a specific cortical area (the extrastriate body area, or EBA). Several studies, including the initial report, observed a high degree of overlap between EBA and both LOC and MT (Downing et al., 2001; Spiridon et al., 2006; Downing et al., 2007). Moreover, functional effects reported within EBA are also observed within MT+ and LOC (Astafiev et al., 2004), to the extent that these areas also show a preference for body parts over objects (i.e., the conventional localizer for EBA; Spiridon et al., 2006; Downing et al., 2007). Comparison of our own data with ROIs created by another group from an independent group of subjects (Julian et al., 2012) showed almost complete overlap of EBA with MT+ (see Appendix). At present, it is not possible to speculate about the relationship of EBA with MTc because it is not clear to what extent area EBA exists outside of LOC and MT, and the functional properties of MTc remain inadequately defined in both macaques and humans. However, as illustrated in Fig. 3, future fMRI experiments at high spatial resolution might well clarify these relationships.

### Implications

In the human fMRI literature, LO and MT+ are among the most important and often-studied areas in mid-level visual cortex. EBA and the nearby within-area patches have an additional literature of their own. Hence it is somewhat surprising that the interpretive problem raised by this reported mutual spatial overlap has not been more explicitly discussed, nor addressed experimentally. One possible explanation is that many studies have localized and focused on just one or two of these areas; overlapping area(s) were of little specific interest. Thus sometimes, one is left wondering about the extent to which prior ROI data from “MT” or “LO” or “EBA” in the current literature were partially sampled from one or more overlapping region. Thus if/when this spatial overlap can be explained, it will clarify the functional organization of human visual cortex in a fundamental way.

## Appendix

**Figure:**
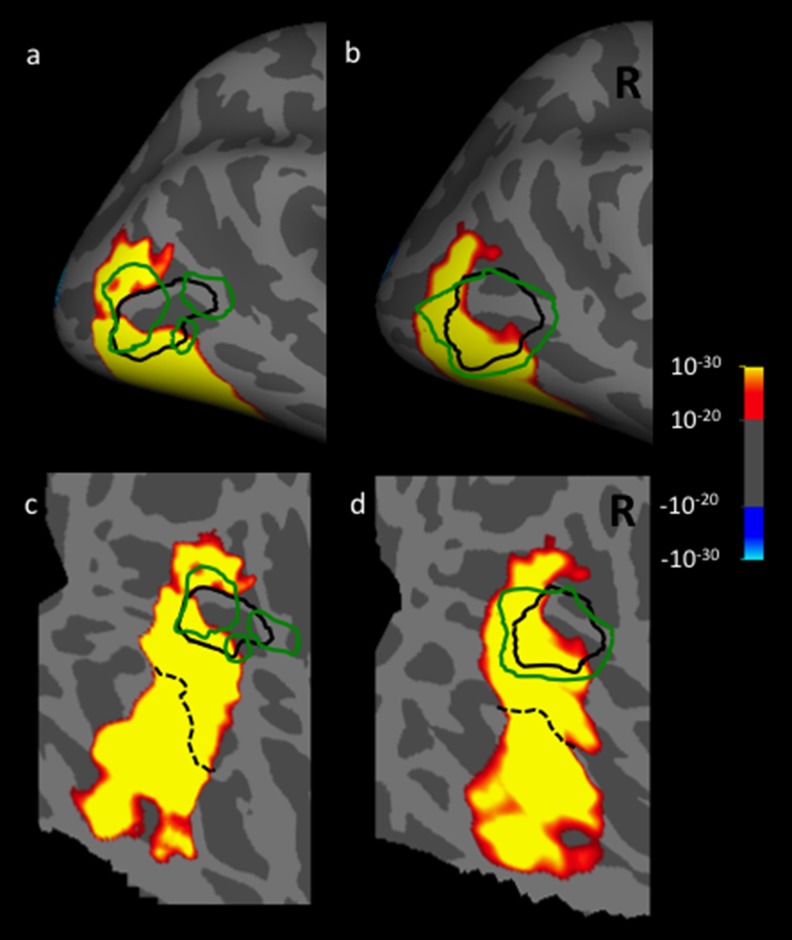
Location of the extrastriate body area (EBA), relative to the borders of LO and MT+, defined independently in a common cortical surface. The border of EBA (indicated with a green line) was defined by a previous study conducted by another group (Julian et al., 2012), in which activity evoked by movies of moving bodies was contrasted with activity evoked by movies of moving objects. That data (*n* = 30) was then used to define area EBA using a group-constrained subject-specific analysis. Here, that localization of EBA is overlaid on our data, which was generated in a different set of subjects (*n* = 13), using different localizers for LO and MT+ (see Fig. 1). In this post-hoc comparison, EBA overlaps MT+, essentially completely.
